# Effect of Soil Temperature on Reproduction of Root-knot Nematodes in Flue-cured Tobacco with Homozygous *Rk1* and/or *Rk2* Resistance Genes

**DOI:** 10.2478/jofnem-2023-0032

**Published:** 2023-08-01

**Authors:** Jill R. Pollok, Charles S. Johnson, J.D. Eisenback, T. David Reed, Noah Adamo

**Affiliations:** Virginia Tech, Southern Piedmont Agricultural Research and Extension Center, 2375 Darvills Rd, Blackstone, VA 23824; Virginia Tech, School of Plant and Environmental Sciences, Blacksburg, VA 24060

**Keywords:** *Meloidogyne incognita* race 3, *Meloidogyne arenaria*, *Nicotiana tabacum*, reproductive index, plant disease loss

## Abstract

Most commercial flue-cured tobacco cultivars contain the *Rk1* resistance gene, which provides resistance to races 1 and 3 of *Meloidogyne incognita* and race 1 of *M. arenaria*. A number of cultivars now possess a second root-knot resistance gene, *Rk2*. High soil temperatures have been associated with a breakdown of root-knot resistance genes in a number of crops. Three greenhouse trials were performed from 2014 to 2015 investigate the effect of high soil temperature on the efficacy of *Rk1* and/or *Rk2* genes in reducing parasitism by a population of *M. incognita* race 3. Trials were arranged in randomized complete block design in open-top growth chambers set at 25°, 30°, and 35°C. Plants were inoculated with 3,000 eggs and data were collected 35 days post-inoculation. Galling, numbers of egg masses and eggs, and reproductive index were compared across cultivar entries. Nematode reproduction was reduced at 25°C and 30°C on entries possessing *Rk1* and *Rk1Rk2* compared to the susceptible entry and the entry possessing only *Rk2*. However, there were often no significant differences in reproduction at 35°C between entries with *Rk1* and/or *Rk2* compared to the susceptible control, indicating an increase of root-knot nematode parasitism on resistant entries at higher temperatures. Although seasonal differences in nematode reproduction were observed among experiments, relative differences among tobacco genotypes remained generally consistent.

Flue-cured tobacco (*Nicotiana tabacum* L.) is an important agricultural crop grown throughout the world (Johnson and Chinheya, 2018). Root-knot nematodes (*Meloidogyne* spp.) are a common pathogen wherever tobacco is grown, and *M. incognita* (Kofoid and White, 1919) Chitwood, 1949, *M. arenaria* (Neal, 1889) Chitwood, 1949, and *M. javanica* (Treub, 1885) Chitwood, 1949 are the three root-knot nematode species of economic importance regularly found in flue-cured tobacco (Johnson et al., 2005). Utilizing host plants with nematode resistance is a common control strategy for root-knot nematodes (Koenning et al., 1999). In Virginia, most commercial flue-cured tobacco cultivars possess the *Rk1* resistance gene, which provides resistance to races 1 and 3 of *M. incognita* and race 1 of *M. arenaria* (Ng’ambi et al., 1999a). An increasing number of cultivars also possess a second root-knot nematode resistance gene, *Rk2* (Reed et al., 2015), which confers some degree of resistance or tolerance to *M. javanica* (Schweppenhauser, 1968, 1975; Ternouth et al., 1986). Due to widespread use of tobacco cultivars with the *Rk1* gene, root-knot nematode species and races that cannot be controlled by cultivars with *Rk1* are increasing in prevalence and importance, including *M. incognita* races 2 and 4, *M. arenaria* race 2, and *M. javanica* (Fortnum et al., 1984; Barker, 1989).

Soil temperature plays a critical role in the efficacy of resistance genes in a number of crops. One of the most studied of these temperature-nematode resistance-gene interactions involved the *Mi* gene in tomato. Dropkin (1969) observed that only 2% of root-knot juveniles developed in the roots of tomato plants containing the *Mi* resistance gene at 28°C, but 87% developed at 33°C. In the common bean (*Phaseolus vulgaris* L.), resistance to *M. incognita* due to recessive genes failed at 26°C to 28°C, while resistance due to dominant genes failed in plants at 28°C to 30°C (Omwega and Roberts, 1992). The rate of nematode development and total population numbers increased on *M. incognita*-resistant sweet potato (*Ipomea batata* L.) entry ‘Nemagold’ as temperatures increased from 24°C to 32°C (Jatala and Russell, 1972). In grape rootstocks, resistance to *Meloidogyne* spp. Began to break down at 27°C with increased root galling and egg mass formation (Ferris et al., 2013).

Root-knot resistance does not always fail at higher temperatures in tomatoes. Ammati et al. (1986) noted that root-knot resistance failed at higher temperatures in some plants but not others. Tomato entry ‘VFN8,’ containing the *Mi* resistance gene, was resistant to *M. incognita* at 25°C and susceptible at 32°C. Three other genotypes retained resistance at 32°C. A suggested explanation for this occurrence was that gene(s) other than the *Mi* gene may have been present and they may have preserved resistance in those genotypes (Ammati et al., 1986).

Baum et al. (1995) noted resistance to *M. incognita* race 3 starting to break down at 28°C in the flue-cured tobacco cultivar NC 95 containing the *Rk1* gene. Increased galling and numbers of egg masses were observed at 28°C, with further increases noted at 31°C and 35°C. Schweppenhauser (1968) observed greater *M. javanica* infection on resistant (*Rk2* gene) and susceptible tobacco lines in summer compared to winter in Zimbabwe, and assumed this apparent trend was due to approximate greenhouse air temperatures of up to 41°C in summer, in contrast to 21°C to 35°C in winter. Shepherd and Barker (1990) also noted increased root invasion and galling on resistant entries by *M. javanica* when soil temperature increased from 25°C to 30°C, yet when soil temperature was raised again to 35°C, root invasion was diminished; however, it was not clear whether the *M. javanica*-resistant entries contained *Rk2*. A report from Japan also noted root galling, rate of invasion, and nematode development by *M. javanica* increased on the tomato cultivar Okinawa compared to susceptible cultivars as temperature increased from 28°C to 35°C, yet the mechanism of resistance in this entry is unclear (Fukudome and Kamigama, 1982). These studies noted changes in resistance to *Meloidogyne* spp. associated with elevated temperatures. Research on the effect of temperature on *Rk2* specifically, alone or combined with *Rk1,* has been limited. The objective of this study was to compare effects of increasing soil temperature on resistance against a population of *M. incognita* race 3 conferred by *Rk2*, *Rk1*, or both genes in combination.

## Materials and Methods

*Population source:* A population of root-knot nematodes was received from Clemson University in Clemson, South Carolina and identified as *M. incognita* race 3 based on perineal pattern and stylet morphology, esterase phenotypes, species-specific sequence-characterized-amplified-region (SCAR) primers (MiF/MiR, IncK14F/R, Rinc/Rinc), PCR-DNA sequences on rRNA 18S, ITS, 28S, D2/D3, histone and mitochondrial DNA COII-16S gene (W. M. Ye, pers. comm.), and three greenhouse differential host trials to identify to a host race level (Taylor and Sasser, 1978). The population was maintained on susceptible tomato (*Solanum lycopersicum* L.) cultivar ‘Rutgers’ in greenhouses at the Southern Piedmont Agricultural Research and Extension Center (SPAREC) near Blackstone, VA.

*Greenhouse trials:* Nine greenhouse trials were conducted in 2014 and 2015 to evaluate the influence of temperature on root-knot resistance gene efficacy in flue-cured tobacco. Among the nine trials, three were performed during the summer (June–July 2014), three were performed during the fall (September–October 2014), and were performed during the winter (December 2014–January 2015). All trials were conducted at the SPAREC and organized in a randomized complete block design. Six tobacco entries were evaluated in each trial: the cultivar Coker 371-Gold (C371G), susceptible to four common root-knot nematode species (*M. incognita*, *M. arenaria*, *M. javanica*, and *M. hapla* Chitwood, 1949); cultivars SC 72 and NC 95, homozygous for *Rk1*; the breeding line T-15-1-1, homozygous for *Rk2*; and the breeding lines STNCB-2-28 and NOD 8, homozygous for both *Rk1* and *Rk2*. Plants were grown in adjacent open-top root zone cabinet growth chambers (Environmental Growth Chambers, Chagrin Falls, OH) set at one of the three soil temperatures so that nematode reproduction could be investigated at 25 ± 1.2°C, 30 ± 1.6°C, and 35 ± 2.3°C. Soil temperatures were taken with a digital stem thermometer between 8 a.m. and 9 a.m., and again between 4 p.m. and 5 p.m. each day, and the temperature range was averaged across all experiments (General Tools & Instruments LLC, Secaucus, NJ). The six entries were replicated four times in the three summer trials (one trial at each soil temperature) and six times in each of the three fall and three winter trials. Each seedling was transplanted into a 10-cm diameter clay pot filled with a 2:1 mixture of topsoil (53% sand, 40% silt, 7% clay; pH 5.5) and Profile® Greens Grade™ porous ceramic material (Profile Products, Buffalo Grove, IL) when seedlings had formed approximately four true leaves (approximately 5–10 cm tall). Each plant was inoculated with 3,000 root-knot nematode eggs one week after transplanting by pipetting 40 ml of egg suspension calibrated to contain 75 eggs/ml into two holes approximately 4 cm deep on either side of the plant. Plants were grown without supplemental lighting in summer and fall trials, but plants in the winter trials were grown with supplemental lights set to a 13:11 light:dark photoperiod using GE Plant and Aquarium Ecolux 40 W wide spectrum bulbs (General Electric, USA). Soil moisture was standardized between chambers based on a soil moisture deficit in a designated pot in each growth chamber using a soil tensiometer system (Irrometer Co. Inc., Riverside, CA).

Data on percent root galling, number of egg masses per gram of root, and number of eggs per gram of root were collected after 35 days. Soil was rinsed from roots and the fresh root system was weighed. Galled roots were separated from non-galled roots and percent root galling was calculated based on fresh weight of galled roots versus fresh weight of the entire root system. Galled roots were then recombined with non-galled roots and mixed. Mixed galled and healthy roots were then divided in half by weight, and half were stained with 0.15 g/L Phloxine B (Dickson and Struble, 1965) to define egg masses. Numbers of egg masses from three stained 1 g subsamples per plant were counted using a dissecting microscope at x10 to estimate the number of females. Eggs were bleach-extracted from the second half of the root system (Hussey and Barker, 1973). Extracted eggs were suspended in 500 ml of water and counted in two 10 ml aliquots using a compound microscope at x40 magnification. To assess nematode reproductive capability on each entry, reproductive index (P_f_/P_i_) was calculated by dividing the final number of eggs extracted per plant (P_f_) by the number of eggs in the initial inoculum (P_i_) (Sasser et al., 1984).

*Statistical analysis:* Data from each trial were analyzed separately by a one-way analysis of variance (ANOVA); means were compared using the Tukey-Kramer honest significant difference (HSD) test (*P* = 0.05). Percent galling, egg mass counts, and egg counts were transformed (log_10_ (x + 1)) before ANOVA. Non-transformed means were presented. Data were analyzed using the Statistical Analysis System-JMP® Pro 11 (SAS Institute, Cary, NC).

## Results

**Figure 1:**
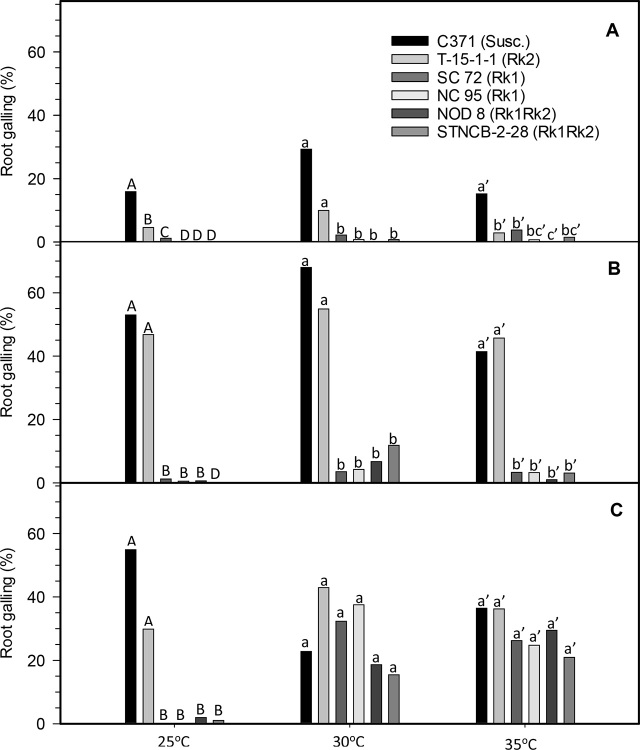
Percent root galling of six tobacco (*Nicotiana tabacum* L.) entries inoculated with a population of *Meloidogyne incognita* race 3 and grown at three soil temperatures in greenhouse trials. (A) summer (June–July 2014), (B) fall (Sept–Oct 2014), and (C) winter (Dec 2014–Jan 2015). Data presented are the means of four observations from the summer trial and six observations from the fall and winter trials. Means for experimental entries associated with the same letter(s), within each season and soil temperature, are not significantly different according to the Tukey-Kramer honest significant difference (HSD) test of transformed [log_10_(x+1)] data (*P* = 0.05). Susc. = susceptible.

**Figure 2:**
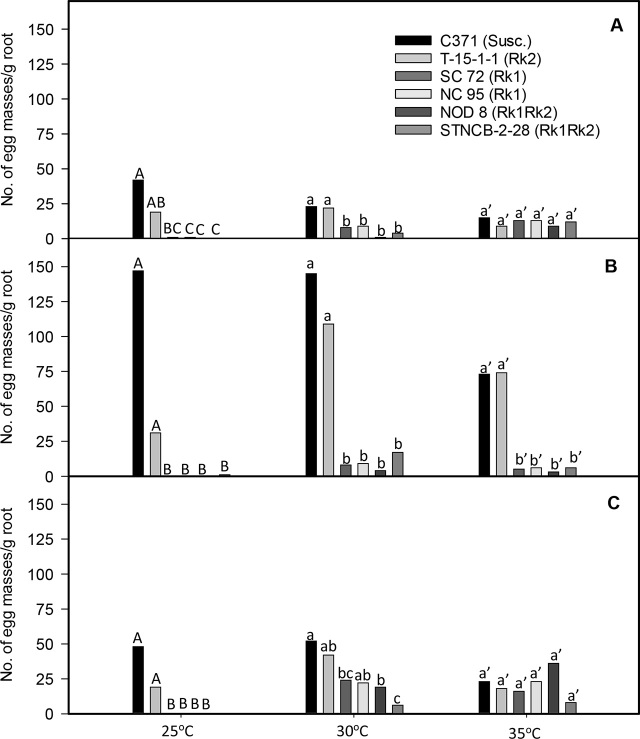
Numbers of egg masses per gram root of a population of *Meloidogyne incognita* race 3 on six tobacco (*Nicotiana tabacum* L.) entries grown at three soil temperatures in greenhouse trials. (A) summer (June–July 2014), (B) fall (Sept–Oct 2014), and (C) winter (Dec 2014–Jan 2015). Data presented are means of four observations from the summer trial and six observations from the fall and winter experiments. Means for experimental entries associated with the same letter(s), within each season and soil temperature, are not significantly different according to the Tukey-Kramer honest significant difference (HSD) test of transformed [log_10_(x+1)] data (*P* = 0.05). Susc. = susceptible.

**Figure 3:**
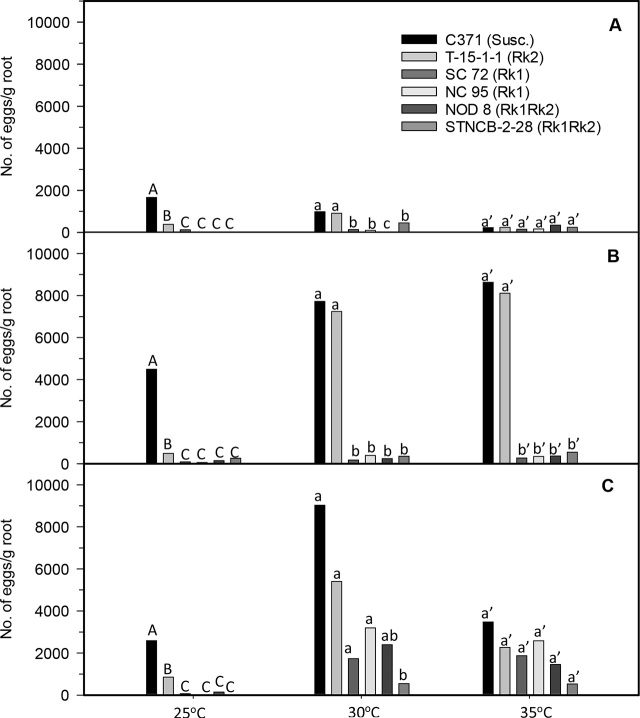
Total egg production per gram of root of a population of *Meloidogyne incognita* race 3 on six tobacco (*Nicotiana tabacum* L.) entries grown at three soil temperatures in greenhouse trials. (A) summer (June–July 2014), (B) fall (Sept–Oct 2014), and (C) winter (Dec 2014–Jan 2015). Data presented are means of four observations from the summer trial and six observations from the fall and winter experiments. Means for experimental entries associated with the same letter(s), within each season and soil temperature, are not significantly different according to the Tukey-Kramer honest significant difference (HSD) test of transformed [log_10_(x+1)] data (*P* = 0.05). Susc. = susceptible.

*Rk1 entry results:* In all trials, root galling and numbers of egg masses and eggs per gram of root were significantly lower at 25°C in both entries possessing only *Rk1* compared to the susceptible control, C371G (*P* ≤ 0.05; Figs. 1–3). Galling and numbers of egg masses and eggs were also reduced on both entries possessing *Rk1* alone versus the susceptible control at 30°C in the summer and fall trials (*P* ≤ 0.05), but not in winter. In contrast, no differences in galling were noted among any of the entries at 30°C or 35°C in the winter test (Fig. 1C). Egg mass numbers were also similar for the *Rk1* entries and the susceptible control at 35°C in the summer trial and at 30 and 35°C in the winter trial (Figs. 2A, 2C). Total numbers of eggs were lower at 25°C on *Rk1* entries versus the susceptible control in all trials (*P* ≤ 0.05; Fig. 3). Total numbers of eggs were also similarly lower on both of the *Rk1* entries relative to the susceptible control at 30°C in the summer and fall experiments (Figs. 3A, 3B). In the winter study, egg numbers were lower at 30°C on the *Rk1* entry SC 72 versus the susceptible control but not on the *Rk1* entry NC 95 (Fig. 3C). At 35°C, total egg numbers were lower on both *Rk1* entries compared to the susceptible control in the fall trial (Figs. 3B). However, the total number of nematode eggs were not lower on the *Rk1* entries compared to the susceptible control in the summer and winter trials, when nematode egg numbers appeared relatively low, even on the susceptible control (Figs. 3A, 3C). Nematode reproductive index was significantly lower on *Rk1* entries compared to the susceptible entry at both 25°C and 30°C in all trials (*P* ≤ 0.05; Table 1). At 35°C, nematode reproductive index was significantly lower on the *Rk1* entries versus the susceptible control only in the fall trial, but not in the summer or winter trials (Table 1).

**Table 1.** Reproduction of a population of *Meloidogyne incognita* race 3 on six tobacco (*Nicotiana tabacum* L.) entries grown at three soil temperatures in greenhouse trials conducted in 2014–2015.

**Plant entry**	**Resistance genes**	**Soil temperature (°C)**	**Reproductive index (P_f_/P_i_)^ab^ by trial**		

			**June–July 2014 (summer)**	**Sept–Oct 2014 (fall)**	**Dec 2014–Jan 2015 (winter)**
C371G	None	25	11.3 A	18.5 a	9.4 a’
T-15-1-1	*Rk2*	25	3.0 B	1.9 b	3.6 b’
SC 72	*Rk1*	25	0.7 C	0.3 b	0.2 c’
NC 95	*Rk1*	25	0.6 C	0.2 b	0.1 c’
NOD 8	*Rk1Rk2*	25	0.2 C	0.4 b	0.2 c’
STNCB-2-28	*Rk1Rk2*	25	0.2 C	0.5 b	0.1 c’
C371G	None	30	14.8 A	29.3 a	38.9 a’
T-15-1-1	*Rk2*	30	11.8 A	35.4 a	25.3 a’
SC 72	*Rk1*	30	1.9 B	0.7 b	3.8 bc’
NC 95	*Rk1*	30	1.3 B	1.2 b	8.7 b’
NOD 8	*Rk1Rk2*	30	0.1 B	0.9 b	3.3 bc’
STNCB-2-28	*Rk1Rk2*	30	1.7 B	1.2 b	1.8 c’
C371G	None	35	3.5 A	50.0 a	10.5 a’
T-15-1-1	*Rk2*	35	3.5 A	37.0 a	10.8 a’
SC 72	*Rk1*	35	2.3 A	1.1 b	3.4 a’
NC 95	*Rk1*	35	1.9 A	1.7 b	5.8 a’
NOD 8	*Rk1Rk2*	35	3.7 A	1.2 b	3.2 a’
STNCB-2-28	*Rk1Rk2*	35	2.9 A	2.5 b	2.5 a’

a Reproductive index = final population/initial population (P_f_/P_i_).

b Data presented are the means of four observations from the summer trial and six observations from the fall and winter experiments.

Means for experimental entries associated with the same letter(s), within each season and soil temperature, are not significantly different according to the Tukey-Kramer honest significant difference (HSD) test of transformed [log_10_(x+1)] data (*P* = 0.05). Susc. = susceptible.

Similar trends were observed in galling in the summer and fall trials, and in reproduction in the fall trial at 35°C, but not in the winter trials (Fig. 1, Table 1). Significant differences were observed in numbers of egg masses and eggs among entries at 35°C in the fall trials, but not in the summer or winter experiments (Figs. 2, 3).

*Rk2 entry results:* Root galling in the summer trial was lower on all entries possessing the *Rk2* gene compared to susceptible C371G when soil temperature was 25°C or 35°C (*P* ≤ 0.05), but not 30°C (Fig. 1). *Rk2* did not reduce galling compared to C371G at any soil temperature in the fall and winter trials. At 25°C, fewer egg masses were observed per gram of root on T-15-1-1 versus the susceptible control in both fall and winter trials (*P* ≤ 0.05; Fig. 2). No differences were observed between the *Rk2* entry and the susceptible control at 25°C in the summer trial, or at 30°C or 35°C in any trial. Total numbers of eggs per gram of root were similar for the *Rk2* entry and the susceptible control at all three temperatures in all trials, with the exception that fewer eggs were counted for T-15-1-1 than for C371G at 25°C in the fall study (*P* ≤ 0.05; Fig. 3). While reproductive indices were significantly lower for *Rk2* plants relative to the susceptible control at 25°C in all experiments (*P* ≤ 0.05), reproductive indices of these entries were more similar at 30°C and 35°C (Table 1).

Both galling and numbers of egg masses were significantly lower on the two *Rk1* entries compared to the *Rk2* entry at 25°C in all trials (*P* ≤ 0.05; Figs. 1, 2). At 30°C, these differences were significant only in the summer and fall experiments (*P* ≤ 0.05; Figs. 1, 2). At 35°C, both galling and egg mass numbers in the fall experiment were lower on the *Rk1* entries compared to susceptible C371G, but not in the summer or fall trials (*P* ≤ 0.05; Figs. 1, 2). Total numbers of nematode eggs per gram of root were lower for *Rk1* entries versus the *Rk2* entry at 25°C in the winter trial (*P* ≤ 0.05; Fig. 3). Total nematode egg numbers in the fall 25°C test were lower for only one *Rk1* entry, NC 95, compared to the *Rk2* entry. Significantly fewer eggs were recovered from *Rk1* entries compared to the *Rk2* entry at 30°C in both summer and fall tests, but not in the winter test (*P* ≤ 0.05; Fig. 3). In contrast, differences in total egg numbers between the *Rk1* entries and C371G at 35°C were only significant in the fall test, but not in the summer or winter trials (*P* ≤ 0.05; Fig. 3). Reproductive indices at 25°C were lower for *Rk1* entries relative to the *Rk2* entry in the fall and winter trials, but not in the fall trial (Table 1). Reproductive indices at 30°C were significantly lower on *Rk1* entries compared to the *Rk2* entry T-15-1-1 in all trials (*P* ≤ 0.05; Table 1). Reproductive indices at 35°C were significantly lower on *Rk1* entries versus the entry with only *Rk2* in the summer, fall, and winter trials. (Table 1).

*Rk1Rk2 entry results:* Galling remained significantly lower (*P* ≤ 0.05) for entries with both *Rk1* and *Rk2* than for the susceptible control at all temperatures in the summer and fall experiments. This also held true at 25°C in the winter trial, but galling was similar to that on all other entries, including the susceptible control, at 30°C and 35°C in the winter trial (Fig. 1). Galling was similar when *Rk2* was combined with *Rk1* in comparison to *Rk1* alone at all temperatures in every trial, except that galling was lower on NOD 8 compared to SC 72 at 35°C in the summer trial (*P* ≤ 0.05; Fig. 1). Galling on entries with both *Rk1* and *Rk2*, relative to *Rk2* alone, was also consistently lower at 25°C across all experiments, and was also lower at 30°C for both entries possessing both resistance genes in the summer and fall trials, but not in the winter test. Possession of both *Rk1* and *Rk2* reduced galling at 35°C versus only *Rk2* in the fall trial, but not in the winter trial. Galling was significantly lower for NOD 8 versus the *Rk2*-only entry in the summer trial but was similar for both STNCB-2-28 and the *Rk2*-only entry in that experiment (Fig. 1).

Mean egg mass numbers per g of root were always significantly lower for entries with both *Rk1* and *Rk2* than for the susceptible control and T-15-1-1 (with *Rk2* alone) at 25°C (*P* ≤ 0.05), and at 30°C in summer and fall trials (*P* ≤ 0.05; Fig. 2). In the winter trial, mean egg mass numbers were significantly lower at 30°C on STNCB-2-28, but not for NOD 8, compared to the susceptible control (*P* ≤ 0.05; Fig. 2). Mean egg mass numbers were similar among all entries at 35°C in the summer and winter trials but were lower for entries with both *Rk1* and *Rk2* compared to the susceptible control and the entry with *Rk1* alone in the fall study. At 25°C and 35°C, mean egg mass numbers were similar in all tests among entries possessing both *Rk1* and *Rk2* as well as those containing only *Rk1*. Mean egg mass numbers were similar at 30°C among entries with *Rk1*, with or without *Rk2*, in the fall trial, but were significantly lower (*P* ≤ 0.05) on NOD 8, but not STNCB-2-28 in the summer experiment. They were significantly lower on STNCB-2-28, but not NOD 8, in the winter study (Fig. 2).

Mean total numbers of nematode eggs produced per gram of feeder root were lower at 25°C for both *Rk1Rk2* entries versus both the susceptible control and the entry possessing only *Rk2* in all trials (Fig. 3; *P* ≤ 0.05). Mean numbers of eggs per g of root at 30°C were again lower in the fall experiment for both *Rk1Rk2* entries compared to both the fully susceptible control and the entry possessing only *Rk2* (*P* ≤ 0.05), but were similar among STNCB-2-2, the *Rk2* entry, and the susceptible control in the summer trial. In the winter experiment, mean numbers of eggs/g root at 30°C were lower for the *Rk1Rk2* entry STNCB-2-28 versus susceptible C 371G and the *Rk2* entry T-15-1-1 (*P* ≤ 0.05), but similar for the other *Rk1Rk2* entry NOD 8, and T-15-1-1. At 35°C, mean numbers of total eggs produced per gram of root were again significantly lower on both *Rk1Rk2* entries compared to susceptible C371G and T-15-1-1, but no statistically significant differences were observed among any of the entries in the summer and winter studies, when very few nematode eggs were observed, even on the susceptible control C371G (Fig. 3).

Nematode egg production was similar among the *Rk1Rk2* entries and the *Rk1*-only entries in all three trials at 25°C, and at 30°C in the fall experiment (Fig. 3). Total nematode eggs/g of root were low, but still intermediate for the *Rk1Rk2* entry STNCB-2-28 versus all of the other entries except *Rk1Rk2* entry NOD 8 at 30°C in the summer trial. Total nematode egg production/g of root at 30°C in the winter trial was lower on *Rk1Rk2* entry STNCB-2-28 compared to the *Rk1* entry NC 95 and the *Rk1Rk2* entry NOD 8, but was similar for NOD 8 compared to both entries that possessed only *Rk1* – SC 72 and NC 95. No differences were observed among entries that possessed *Rk1* at 35°C in any of the three studies, whether or not *Rk2* was also present. (Fig. 3).

Reproductive indices were lower versus the susceptible C371G on entries possessing only *Rk1* at all temperatures, in all trials, except at 35°C in the winter screen, when numerical differences among entries were not statistically significant (*P* ≤ 0.05; Table 1). On the other hand, similar differences between fully susceptible C371G and the *Rk2* entry T-15-1-1 were only significant at 25°C, but never at 30°C or 35°C. Reproductive indices were always numerically lower at 25°C for the two *Rk1* entries compared to T-15-1-1. These differences were significant in the summer and winter experiments, but not in the study conducted during the fall. Similar differences were also observed in all trials conducted at 30°C, and in the fall trial at 35°C, but no significant differences were observed across all entries at 35°C in the summer and winter studies. Reproductive indices were similar among entries possessing *Rk1* either alone or with *Rk2* at all temperatures and in all experiments, with the exception of the fall trial at 30°C, when reproductive indices were higher for the *Rk1Rk2* entry STNCB-2-29 than for the *Rk1* entry NC 95. Reproductive indices were also always numerically lower at 25°C when *Rk1* was combined with *Rk2*, although the difference in the fall study was not statistically significant (Table 1). Possession of both root-knot resistance genes versus only *Rk2* was always associated with lower reproductive indices when investigations were performed at 30°C, but only in the fall test at 35°C (*P* ≤ 0.05; Table 1).

## Discussion

Results from these studies suggested that resistance to *M. incognita* race 3 declined at 30°C, and especially at 35°C, compared to 25°C. A drastic reduction in nematode reproduction at 25°C and 30°C was associated with *Rk1* alone and *Rk1Rk2* together compared to susceptible cultivar C371G. However, reproduction on both *Rk1* and *Rk1Rk2* appeared similar to that on C371G at 35°C. Additionally, *Rk1Rk2* did not appear to be significantly more effective than *Rk1* in suppressing reproduction of this population of *M. incognita* race 3. No consistent trends between *Rk1* and *Rk1Rk2* occurred with regard to production of egg masses or total nematode eggs, nor reproductive index, across temperatures, perhaps because there was so little parasitism on these cultivars and breeding lines.

The temperature-sensitive *Rk1* resistance results corroborate those by Baum et al. (1995), who observed that *M. incognita* race 3 resistance began breaking down at 28°C, and further at 31°C and 35°C, in the *Rk1*-containing tobacco entry NC 95. Results of this study are also supported by results from other crops. Temperature-sensitive *Meloidogyne* resistance has been noted in tomato with the *Mi* resistance gene (Dropkin, 1969; El-Sappah et al., 2019), common bean (Omwega and Roberts, 1992), sweet potato (Jatala and Russell, 1972), grape (*Vitis* spp.) rootstocks (Ferris et al., 2013), and cotton (*Gossypium hirsutum* L.) (Carter, 1982).

Similarly, numbers of egg masses and eggs, and the reproductive index of plants with *Rk2,* were almost always significantly lower than those of the susceptible control at 25°C. However, this trend seemed to be lost at 30°C and 35°C when there were frequently no significant differences in reproduction between the *Rk2* entry and the susceptible control. The *Rk2* gene appeared to inhibit reproduction less effectively at 30°C and 35°C than at 25°C, although *Rk2* seemed to provide only modest resistance to this population of *M. incognita* race 3, even at 25°C, especially compared to effects of *Rk1* or *Rk1Rk2*.

Schweppenhauser (1968) observed higher *M. javanica* infection in resistant (*Rk2*) and susceptible lines in the summer, when approximate greenhouse air temperatures regularly reached 41°C, compared to winter infection, when temperatures were approximately 21°C to 35°C. Ng’ambi et al. (1999b) observed that the entry Okinawa did not provide any resistance to *M. javanica* in their study at greenhouse temperatures of 27 ± 2°C and 30 ± 3°C. In contrast, Fukudome and Kamigama (1982) found that galls caused by *M. javanica* were rare on Okinawa at 28°C, but at 35°C there were no differences in galling, invasion rate, and development compared with the susceptible entry. In Zimbabwe, increased *M. javanica* root invasion and galling on both resistant and susceptible tobacco was observed when soil temperature rose from 25°C to 30°C, but parasitism actually decreased when soil temperature was further increased to 35°C (Shepherd and Barker, 1990). Shepherd and Barker (1990) did not state whether or not *M. javanica*-resistant entries contained the *Rk2* gene, although this seems likely. Despite some variation, this trend toward increased galling at higher temperatures appeared across all entries.

Despite reduced nematode reproduction, galling on the *Rk2* entry in most trials was not significantly different from that of the susceptible entry at all temperatures evaluated. This observation was consistent with results from experiments performed at Virginia Tech in 2010 and 2011 examining *M. javanica* reproduction on the same flue-cured tobacco entries used in the current experiment (Ma et al. unpubl. data). However, Ng’ambi et al. (1999b) noted the amount of galling and reproduction were generally correlated. Consistent with our results, galling by *M. incognita* and *M. javanica* without a corresponding increase in egg masses was noted in *Prunus* rootstocks (Lu et al., 2000). The authors determined that nematodes “can infect and partially develop in resistant root systems, but cannot complete their life cycles.”

Because a reproductive index is calculated as the ratio of the final nematode population relative to the initial population (often calculated from the number of eggs recovered from the root system at the cessation of trials relative to the number of eggs delivered at inoculation), a reproductive index >1 suggests that a plant is not resistant to the nematode. Reproductive indices on the susceptible cultivar and the entry possessing only *Rk2* were always >1 in this research and were generally higher in trials conducted at 30°C or 35°C versus at 25°C. Reproductive indices on entries possessing *Rk1* alone, or with *Rk2,* were always <1 at 25°C but were almost always >1 at 30°C and 35°C, suggesting an inhibition of resistance. Although reproductive indices on *Rk1* and *Rk1Rk2* entries almost always increased from an index of <1 at 25°C to >1 at 30°C and 35°C, reproduction on these entries at these temperatures remained well below that on the susceptible cultivar and the *Rk2* entry in most trials, suggesting that while resistance may diminish with increasing temperature, some effect persists.

A shorter average natural day length (11 hr 42 min) during the fall trial may have affected results compared to the summer (14 hr 25 min) and winter (13 hr) trials (U.S. Naval Observatory). Average natural day length during the winter trial (9 hr 48 min) was supplemented to 13 hr, which could have produced results more similar to those in the summer trial. The explanation for differences in egg mass and egg counts among trials is unclear. Meng et al. (2015) determined light quality had an effect on tobacco seedling root growth, which may have affected root growth in this experiment, but the extent to which this impacted nematode host-seeking and root penetration, as well as subsequent reproduction, is also not clear.

*Meloidogyne incognita* inhabits areas with an average annual temperature range of 18°C to 30°C, with an optimum warm-month temperature of 27°C (Eisenback et al., 1981). In this study, galling and egg mass numbers from the susceptible entry usually decreased as soil temperature increased from 25°C to 35°C, presumably because 35°C is above the optimal temperature range for this population. Soil temperatures in Virginia during the tobacco growing season are commonly above 30°C. Maximum daily soil temperatures logged at the Virginia Tech Southern Piedmont AREC were above 30°C for 8 days in May 2014, 15 days in June and July 2014, 11 days in August 2014, and six days in September 2014. May, June, and July also had maximum temperatures above 35°C for one, two, and three days, respectively. Soil temperatures were taken below grass; soil temperatures in tobacco fields were likely higher.

Fluctuation in soil temperatures might also influence efficacy of resistance to root-knot nematodes in tobacco. Tobacco plants resistant to tobacco mosaic virus (TMV) became susceptible at temperatures above 28°C, but the hypersensitive response returned when temperatures dropped, killing the plant (Malamy et al., 1992; Marathe et al., 2002; Zhang et al., 2009). Tomato plants resistant to *M. incognita* due to the *Mi* gene became susceptible after 48 hr at 34°C and remained susceptible for one to two days after temperatures were reduced to 27°C (Zacheo et al., 1995). Juveniles that had penetrated and started feeding when resistance was deactivated continued developing when temperatures decreased. Some entries of tomato (Ammati et al., 1986; Wang et al., 2013) and pepper (Capsicum annuum L.) (Thies and Fery, 1998; Djian-Caporalino et al., 1999, 2001) retained resistance to *Meloidogyne* species at high temperatures, even up to 32°C and 42°C in four pepper entries (Djian-Caporalino et al., 2001). Perhaps there are sources of temperature-stable root-knot nematode resistance in wild *Nicotiana* relatives that could mitigate some of these issues. However, at present, flue-cured tobacco growers will continue to face challenges associated with high and fluctuating soil temperatures complicating the management of root-knot nematodes.
